# Time Course of Evolution of Disability and Cause‐Specific Mortality After Ischemic Stroke: Implications for Trial Design

**DOI:** 10.1161/JAHA.117.005788

**Published:** 2017-06-11

**Authors:** Aravind Ganesh, Ramon Luengo‐Fernandez, Rose M. Wharton, Sergei A. Gutnikov, Louise E. Silver, Ziyah Mehta, Peter M. Rothwell, the Oxford Vascular Study

**Affiliations:** ^1^ Stroke Prevention Research Unit Nuffield Department of Clinical Neurosciences University of Oxford United Kingdom; ^2^ Health Economics Research Centre Nuffield Department of Population Health University of Oxford United Kingdom

**Keywords:** cerebrovascular disease/stroke, clinical trial design, health economics, longitudinal cohort study, stroke recovery, Ischemic Stroke, Cerebrovascular Disease/Stroke, Epidemiology, Mortality/Survival, Cost-Effectiveness

## Abstract

**Background:**

Outcome in stroke trials is often based on a 3‐month modified Rankin scale (mRS). How 3‐month mRS relates to longer‐term outcomes will depend on late recovery, delayed stroke‐related deaths, recurrent strokes, and nonstroke deaths. We evaluated 3‐month mRS and death/disability at 1 and 5 years in a population‐based cohort study.

**Methods and Results:**

In 3‐month survivors of ischemic stroke (Oxford Vascular Study; 2002‐2014), we related 3‐month mRS to disability (defined as mRS >2) at 1 and 5 years and/or death rates (age/sex adjusted). Accrual of disability and index‐stroke‐related and nonstroke deaths in each poststroke year was categorized according to 3‐month mRS. Among 1606 patients with acute ischemic stroke, 181 died within 3 months, but 126 index‐stroke‐related deaths and 320 other deaths occurred during the subsequent 4866 patient‐years of follow‐up up to 5 years. Although 69/126 (54.8%) post‐3‐month index‐stroke‐related deaths occurred after 1 year, mRS>2 at 1 year strongly predicted these deaths (adjusted hazard ratio=21.94, 95%CI 7.88‐61.09, *P*<0.0001). Consequently, a 3‐month mRS >2 was a strong independent predictor of death at both 1 year (adjusted hazard ratio=6.67, 95%CI 4.16‐10.69, *P*<0.0001) and 5 years (adjusted hazard ratio=2.93, 95%CI 2.38‐3.60, *P*<0.0001). Although mRS improved by ≥1 point from 3 months to 1 year in 317/1266 (25.0%) patients with 3‐month mRS ≥1, improvement in mRS after 1 year was limited (improvement by ≥1 point: 91/858 [10.6%]; improvement to mRS ≤2: 13/353 [3.7%]).

**Conclusions:**

Our results reaffirm use of the 3‐month mRS outcome in stroke trials. Although later recovery does occur, extending follow‐up to 1 year would capture most long‐term stroke‐related disability. However, administrative mortality follow‐up beyond 1 year has the potential to demonstrate translation of early disability gains into additional reductions in long‐term mortality without much erosion by non‐stroke‐related deaths.

**Clinical Perspective**

**What Is New?**

- In the absence of reliable up‐to‐date data on how short‐term outcome after stroke relates to longer‐term outcomes—including late recovery, delayed stroke‐related deaths, recurrent strokes, and nonstroke deaths—we found that functional outcome at 3 months (assessed using the modified Rankin Scale) strongly predicted 5‐year disability and mortality, both overall and in clinically relevant subgroups of treatable major strokes, atrial fibrillation‐related strokes, and lacunar strokes.
- More than a quarter of the deaths beyond 3 months were due to the prior stroke.
- Patients were unlikely to show meaningful recovery beyond 1 year.

**What Are the Clinical Implications?**

- Our results reaffirm the use of the 3‐month modified Rankin Scale as a primary outcome measure in stroke trials. Although later recovery does occur, extending clinical trial follow‐up to 1 year would capture most long‐term stroke‐related disability. However, administrative mortality follow‐up beyond 1 year has the potential to demonstrate translation of early disability gains into additional reductions in long‐term mortality without much erosion by non‐stroke‐related deaths.
- These findings imply that treatments that reduce acute stroke severity (eg, thrombolysis and thrombectomy) or that prevent disabling strokes (eg, anticoagulation for atrial fibrillation) are likely to promote sustained independence and long‐term survival.

## Introduction

Acute stroke treatments sometimes involve a trade‐off between a reduction in 3‐month poststroke disability and an increase in 7‐day mortality.^1^, ^2^ The extent to which the increase in early mortality is further offset by recovery from disability or reductions in mortality after 3 months remains uncertain.^3^ There may be magnification of treatment‐associated differences in mortality from stroke‐related causes beyond the acute period, but there may also be erosion from non‐stroke‐related deaths. Poststroke disability will be similarly affected by late recovery versus subsequent accrual of non‐stroke‐related disability. Longer‐term follow‐up of IST‐3 (the Third International Stroke Trial) showed that the reduction in 6‐month disability seen with thrombolysis was still present at 18 months follow‐up^3^ and that thrombolysis reduced mortality from 3 to 12 months of follow‐up, which difference was maintained at 3 years,^4^ suggesting that follow‐up beyond 3 to 6 months could be informative in other acute stroke trials. However, recommendations for stroke trial design have been inconsistent regarding the appropriate duration of follow‐up. The second STAIR (Stroke Treatment Academic Industry Roundtable) encouraged follow‐up beyond 3 months to evaluate late treatment effects,^5^ as did NINDS (the National Institute of Neurological Disorders and Stroke),^6^, ^7^ but the most recent STAIR even proposed that a 7‐day assessment might be preferable as a primary outcome measure, given the risk of “contamination” by non‐stroke‐related morbidity.^8^

Cohort studies have suggested an association between poststroke functional status and long‐term mortality, but some were limited by retrospective design^9^ and others by possible selection bias,^10^, ^11^, ^12^, ^13^ absence of a consistently timed interview‐based disability assessment, and missing data.^10^, ^12^, ^13^ Moreover, these studies did not establish how many deaths beyond 3 months or 1 year were related to the index stroke and might therefore be preventable by an intervention that reduced severity.

The most commonly used primary outcome measure in acute stroke trials is the modified Rankin Scale (mRS),^14^, ^15^ at least partly because it is simple to administer, has good reproducibility, and avoids major floor or ceiling effects.^16^ Although most functional recovery poststroke happens in the first few months,^17^ recovery beyond 6 months does occur,^18^ but there are few published data on the relationship between early mRS and long‐term disability. Clarifying the time course of evolution of disability and mortality, relative to 3‐month mRS, should inform the optimal durations of both clinical follow‐up in acute stroke trials and data collection on service use, care costs, and utilities for cost‐effectiveness analyses. We therefore determined the associations between 3‐month mRS in ischemic stroke patients and long‐term disability and survival, both overall and in clinically relevant subgroups, and examined the evolution of disability and cause‐specific mortality to 5‐year follow‐up in a population‐based study, OXVASC (the Oxford Vascular Study).

## Methods

### Study Population

The OXVASC population comprises 92 728 patients registered under about 100 primary‐care physicians in 9 general practices across Oxfordshire, United Kingdom. Study methods have been described in detail elsewhere.^19^ Recruitment began in April 2002 and is ongoing. Briefly, multiple overlapping methods of “hot” and “cold” pursuit were used to achieve near‐complete ascertainment of all individuals with transient ischemic attack or stroke. These include: (1) a daily, rapid‐access transient ischemic attack/stroke clinic to which participating general practitioners and the local accident and emergency department refer all individuals with suspected, but not hospitalized, transient ischemic attack or stroke; (2) daily searches of admissions to the medical, cardiology, stroke, neurology, and other relevant wards; (3) daily searches of the local accident and emergency department attendance register; (4) daily searches of in‐hospital death records via the Bereavement Office; (5) monthly searches of all death certificates and coroner's reports for out‐of‐hospital deaths; (6) monthly searches of general practitioner diagnostic coding and hospital discharge codes; (7) monthly searches of all brain and vascular imaging referrals. Direct assessment of rates of ascertainment has shown that it is nearly complete.^20^

### Stroke Diagnosis

Patients were assessed urgently by study clinicians and considered for inclusion. Stroke was diagnosed according to the World Health Organization definition.^21^ Assessments of neurological impairment, presentation, medical and social history, and risk factors were made. Stroke severity was measured using the National Institutes of Health Stroke Scale. All cases were subsequently reviewed by a senior neurologist (P.M.R.) on a daily basis, and imaging results were assessed by the study neuroradiologist. Final classification of transient ischemic attack, stroke, or other condition was made by the same senior neurologist in all cases.

### Patient Follow‐Up and mRS Assessment

Patients were followed‐up face‐to‐face by a study nurse or physician either in a hospital clinic or at home at 1, 3, and 6 months and 1, 5, and 10 years. Recurrent vascular events and functional status (mRS) were recorded at each follow‐up. Raters were all trained in the use of the mRS by use of an instructional DVD with accompanying written materials produced by the University of Glasgow that has previously been used in large‐scale clinical trials.^22^

The mRS is a 7‐point ordinal scale (Table 1) with patients falling into 1 of 7 categories ranging from 0 (no symptoms) to 6 (death). Because our analyses sought to predict death based on 3‐month mRS, patients with a 3‐month mRS of 6 were excluded. Disability at 3 months, 1 year, or 5 years was defined as mRS >2 (score of 3, 4, or 5, excluding 6, ie, death), as is often the case in acute stroke trials,^16^ at the respective follow‐up assessment.

**Table 1:** Categories of the mRS^14^

mRS Score/Category	Description
0	No symptoms at all
1	Able to carry out all usual duties and activities, despite symptoms
2	Unable to carry out all previous activities but able to look after own affairs without assistance
3	Requiring some help but able to walk without assistance
4	Unable to walk without assistance and unable to attend to own bodily needs without assistance
5	Bedridden, incontinent, and requiring constant nursing care and attention
6	Dead

mRS indicates modified Rankin Scale.

Patients who moved out of study area were followed‐up by telephone. Additional information was obtained from a carer in patients with significant impairment of cognition or speech. Recurrent events were also identified by daily OXVASC ascertainment and by review of administrative diagnostic codes from hospital and primary care records.

### Ascertainment of Deaths and Causes of Death

All deaths (with causes) during follow‐up were also recorded from death certificates, coroners' reports, and via the Office for National Statistics National Health Service Central Register. Causes of death were assessed by multiple overlapping sources including autopsy results, clinical details from hospital notes and GP records, and death certificates (further details of death ascertainment in Data S1). Deaths were coded as related to the index stroke if they were deemed either to be directly related to the stroke or were hastened by complications. Study clinicians assigned to code the causes of death did so independently of those performing mRS assessments (without formal blinding) but took recorded specific poststroke impairments into account to decide whether a death was potentially index‐stroke‐related when associated with another direct cause of death, as follows:

1. Aspiration pneumonia: This was coded as index‐stroke‐related if the patient was documented to be at risk of aspiration owing to new dysphagia as a result of the index stroke.
2. Pulmonary embolism: This was coded as index‐stroke‐related if the patient was documented to be newly bed‐ridden or to have markedly restricted mobility following the stroke, and the pulmonary embolus was not clearly associated with another convincing etiology such as cancer or a recent operation.
3. Sepsis from a urinary source (urosepsis): This was coded as index‐stroke‐related if the patient was documented to have new urinary incontinence or bladder dysfunction after the stroke and/or markedly restricted mobility, and the attending physician suspected that this had contributed to the infection.
4. Vascular dementia: This was coded as index‐stroke‐related if the patient was documented to have new cognitive decline following the index stroke, no other cause of dementia was identified, and dementia was recorded as 1 of the causes of death by the attending physician.
5. Fall: This was coded as index‐stroke‐related if the patient was documented to have new impairments in mobility and balance as a result of the index stroke, and the death was the result of a postfall injury (such as a fatal traumatic hemorrhage or fracture).

If the patient had suffered a recurrent stroke before death, then the death was not coded as being index‐stroke‐related owing to the added uncertainty of whether stroke‐related impairments directly causing or hastening death stemmed from the index stroke or the recurrent one.

### Ethical Approval

The study was approved by the Oxfordshire Research Ethics Committee. All strokes were recorded, but only patients consenting to follow‐up, or with assent through a carer if unable to provide informed consent themselves, were included in the analysis.

### Statistical Analyses

Consenting patients recruited from April 2002 to March 2014 and surviving for 3 months after their first stroke in the study period (index stroke) were included in the analysis, to focus on the period beyond 90‐days poststroke that is not conventionally captured in stroke trials. Analyses were censored at 31 July 2015.

The proportions of patients who were (1) disabled, (2) dead/disabled, (3) dead from all causes, and (4) dead from index‐stroke‐related causes at 1, 2, 3, 4, and 5 years were calculated and categorized according to the 3‐month mRS (or the mRS between 1 and 3 months if the 3‐month mRS was missing). Logistic regression was used to adjust the associations of 3‐month mRS and long‐term outcomes for age and sex. Survival to 5 years after index stroke was assessed using Kaplan‐Meier techniques categorized according to 3‐month mRS. Differences in survival in relation to 3‐month mRS scores were assessed using age‐ and sex‐adjusted Cox proportional hazards models.^23^

The above analyses were repeated for the subgroups defined below:

1. *Treatable major strokes or nonhyperacute/minor strokes*: The former were defined as patients seeking medical attention within 6 hours of symptom onset, presenting either to hospital or emergency services with National Institutes of Health Stroke Scale ≥5. These were “minimal criteria” to capture the subset that have been the focus of hyperacute stroke trials and would potentially be eligible for thrombolysis/thrombectomy.^24^, ^25^ The remaining patients were classified as nonhyperacute/minor strokes, to facilitate direct comparison.
2. *Atrial fibrillation (AF)‐related or non‐AF‐related strokes*: The former were defined as patients with a prestroke diagnosis of AF or AF at presentation, meeting criteria for cardioembolic etiology per the TOAST trial (Trial of Org 10172 in Acute Stroke Treatment) classification system.^26^
3. *Lacunar or nonlacunar strokes*: Classified by TOAST criteria for small‐vessel occlusion.^26^

We were specifically interested in examining the long‐term death and/or disability outcomes of AF‐related and lacunar strokes because they have been the focus of several recent stroke trials, particularly of secondary prevention strategies.^27^, ^28^

We also plotted changes in mRS between 3 months and 1 year and between 1 and 5 years after the index stroke for all patients, categorized according to their 3‐month mRS.

Statistical analyses were performed using STATA 13.1 (Statacorp, College Station, TX).

## Results

**Figure 1:**
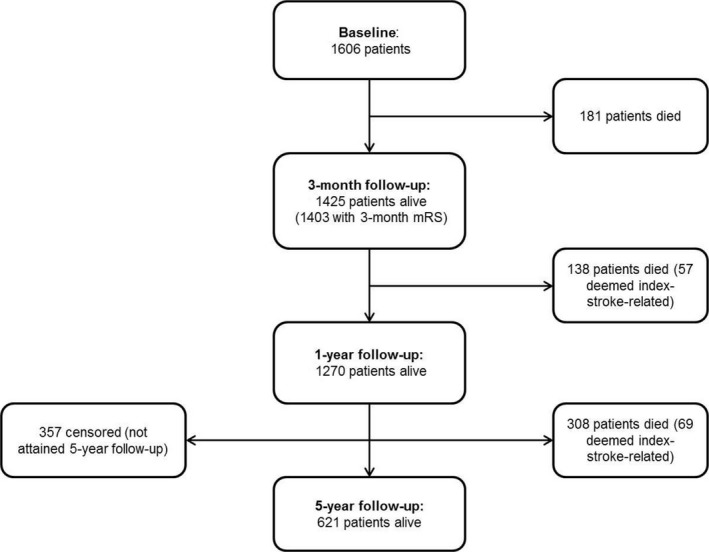
Flowchart illustrating the ischemic stroke patients who were alive at baseline and at 3‐month, 1‐year, and 5‐year follow‐up assessments.

Of 1606 patients with an index acute ischemic stroke, 181 (11.3%) died within 3 months (Figure 1). Baseline characteristics of all 3‐month survivors are shown in Table 2 (subgroups in Table S1). Complete baseline and follow‐up data were available for 1403 (98.5%) of the 1425 3‐month survivors. Of the 22 survivors who were excluded, 18 refused follow‐up, and 4 did not have mRS assessed before 3‐month follow‐up.

**Table 2:** Patient Sample and Characteristics for All 3‐Month Survivors of Ischemic Stroke

	All Stroke Patients (n=1425)
Age, mean (SD), y	73.2 (12.7)
Sex, male (%)	753 (52.8)
Previous history, %	
MI	177 (12.4)
Angina	239 (16.8)
Atrial fibrillation	260 (18.2)
Hypertension	889 (62.4)
Dyslipidemia	469 (32.9)
Diabetes mellitus	205 (14.4)
PVD	108 (7.6)
Stroke	158 (11.1)
TIA	205 (14.4)
Smoking	836 (58.7)
Cancer	22 (15.5)
Prior disability (mRS>2)	244 (17.2)
NIHSS, median (IQR)	2 (1–4)

AF indicates atrial fibrillation; IQR, interquartile range; MI, myocardial infarction; mRS, modified Rankin scale; NIHSS, National Institutes of Health Stroke Scale; PVD, peripheral vascular disease; SD, standard deviation; TIA, transient ischemic attack.

At 5 years, 446 (31.3%) 3‐month survivors were dead, and 255 (26.0%) of those alive were disabled; 357 (25.0%) patients were censored, having not yet attained 5‐year follow‐up, but 1‐year follow‐up data were available for 350 (98.0%) of these patients. There were 126 post‐3‐month index‐stroke‐related deaths and 320 other deaths during 4866 patient‐years of follow‐up from 3‐months to 5‐years. The leading causes of death not related to the index stroke were cancer and cardiac causes (Table 3), accounting for 78 (17.5%) and 71 (15.9%) deaths, respectively, followed by pneumonia (52, 11.7%) and recurrent stroke (44, 9.9%). Aspiration pneumonia was the most common associated cause of post‐3‐month deaths deemed to be related to the index stroke (69, 54.8%).

**Table 3:** Primary Causes of Index‐Stroke‐Related and Non‐Stroke‐Related Death Beyond 90 Days in 3‐Month Survivors of Ischemic Stroke, for Years 1 to 5 Poststroke

Cause of Death (%)	Y 1 (138 Deaths)	Y 2 (85 Deaths)	Y 3 (80 Deaths)	Y 4 (75 Deaths)	Y 5 (68 Deaths)	Overall (446 Deaths)
Index stroke‐related deaths^a^						
Aspiration pneumonia	32 (56.1)	11 (44.0)	13 (59.1)	11 (64.7)	2 (40.0)	69 (54.8)
Vascular dementia	2 (3.5)	2 (8.0)	2 (9.1)	0	1 (20.0)	7 (5.6)
Pulmonary embolism	3 (5.3)	2 (8.0)	0	0	0	5 (4.0)
Urosepsis	2 (3.5)	0	0	0	0	2 (1.6)
Fall	1 (1.8)	0	1 (4.5)	0	0	2 (1.6)
Total stroke‐related deaths	57 (41.3)^b^	25 (29.4)^b^	22 (27.5)^b^	17 (22.7)^b^	5 (7.4)^b^	126 (28.3)^b^
Non‐stroke‐related deaths						
Cancer	22 (15.9)	13 (15.3)	8 (10.0)	16 (21.3)	19 (27.9)	78 (17.5)
Cardiac cause	19 (13.8)	14 (16.5)	17 (21.3)	11 (14.7)	10 (14.7)	71 (15.9)
Pneumonia	10 (7.2)	10 (11.8)	8 (10.0)	13 (17.3)	11 (16.2)	52 (11.7)
Recurrent stroke	15 (10.9)	11 (12.9)	8 (10.0)	3 (4.0)	7 (10.3)	44 (9.9)
Sepsis/infection	4 (2.9)	3 (3.5)	4 (5.0)	2 (2.7)	2 (2.9)	15 (3.4)
Dementia	1 (0.7)	0	4 (5.0)	4 (5.3)	4 (5.9)	13 (2.9)
Peripheral vascular disease	1 (0.7)	2 (2.4)	2 (2.5)	0	1 (1.5)	6 (1.3)
Pulmonary embolism	1 (1.2)	0	0	3 (4.0)	1 (1.5)	5 (1.1)
Chronic lung disease	1 (0.7)	1 (1.2)	1 (1.3)	0	2 (2.9)	5 (1.1)
Renal failure	2 (1.4)	2 (2.4)	1 (1.3)	0	0	5 (1.1)
Gastro‐intestinal bleeding	2 (1.4)	0	0	1 (1.3)	0	3 (0.7)
Fall	0	0	0	1 (1.3)	0	1 (0.2)
Other	3 (2.2)	4 (4.7)	5 (6.3)	4 (5.3)	6 (8.8)	22 (4.9)
Total non‐stroke‐related deaths	81 (58.7)^b^	60 (70.6)^b^	58 (72.5)^b^	58 (77.3)^b^	63 (92.6)^b^	320 (71.7)^b^

Expressed as raw numbers and as percentages of all deaths occurring in that year, listed in descending order of proportion of overall deaths.

a This refers to deaths deemed to be directly related to, or hastened by, the index stroke or its complications (such as immobility), which were not better explained by a recurrent vascular event.

b Under the index‐related stroke section, percentages are in relation to all deaths that year; the offset percentages are in relation to just index‐stroke‐related deaths that year. Percentages in each column (total stroke‐related and non‐stroke‐related deaths) add up to 100%; the various subsections under index‐stroke‐related deaths do not add up to 100% as there were deaths in each year that were deemed to be index‐stroke‐related but did not fall under these specific subsections.

With each subsequent year, index‐stroke‐related deaths accounted for a decreasing proportion of new deaths: 57 (41.3%) deaths from 3 months to 1 year poststroke were deemed to be index‐stroke‐ related, versus only 5 (7.4%) in the fifth year. Although 69/126 (54.8%) post‐3‐month index‐stroke‐ related deaths occurred after 1 year, the mRS at 1 year strongly predicted these subsequent stroke‐ related deaths (age‐ and sex‐adjusted hazard ratio for mRS >2 versus 0‐2=21.94, 95%CI 7.88 to 61.09, *P*<0.0001).

**Figure 2:**
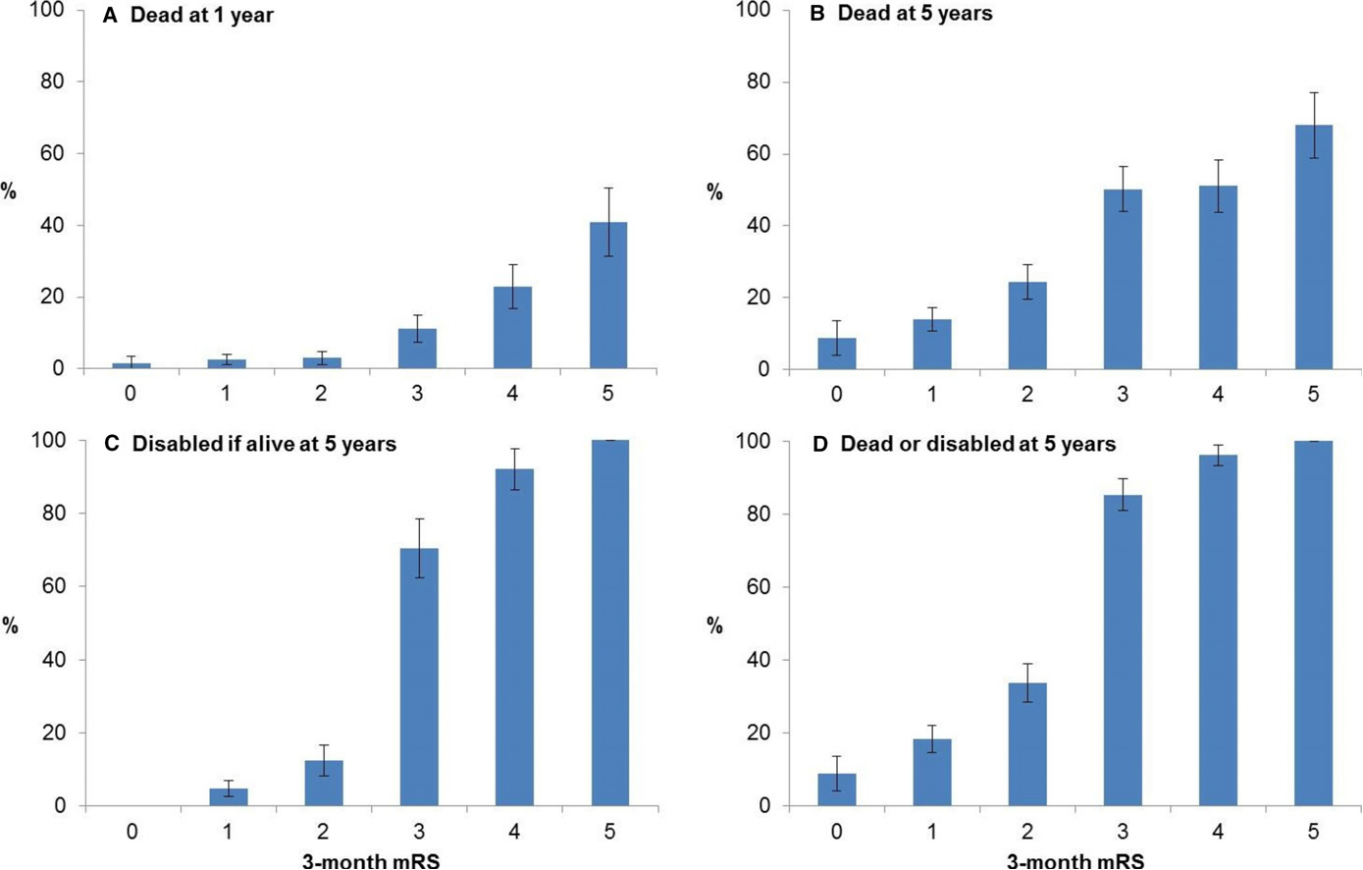
Proportions of 3‐month survivors of ischemic stroke, categorized according to 3‐month mRS, who were (A) dead at 1 year; (B) dead at 5 years; (C) disabled (mRS>2) at 5 years, only including those alive at 5 years in the total; and (D) dead/disabled at 5‐years. Bars represent 95% confidence intervals. mRS indicates modified Rankin Scale.

**Figure 3:**
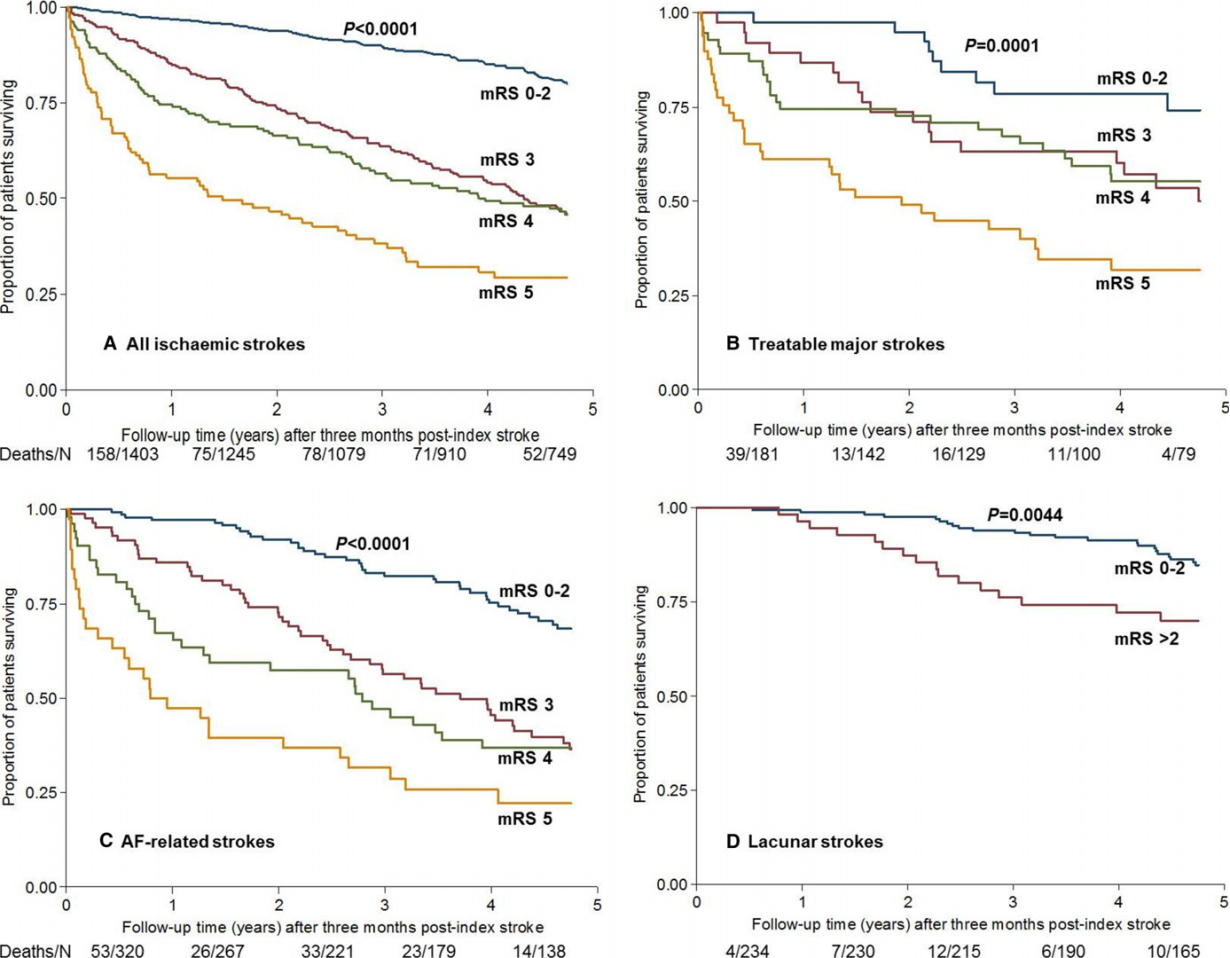
Kaplan‐Meier survival curves after index stroke for 3‐month survivors (3‐month mRS 0‐2 vs 3, 4, 5), for (A) all ischemic strokes, (B) treatable major strokes potentially eligible for thrombolysis/thrombectomy, (C) AF‐related strokes, and (D) lacunar strokes. Because there were few patients with 3‐month mRS>2 for the lacunar group, we compared mRS 0 to 2 vs >2. *P*‐values from log‐rank test for each group are shown. The numbers shown below each panel are all deaths in each time period (Year 0‐1, 1‐2, and so on), over the total number of individuals at risk for that period. AF indicates atrial fibrillation; mRS, modified Rankin Scale.

The 3‐month mRS scores also predicted 1‐ and 5‐year mortality (Figure 2A and 2B). Kaplan‐Meier analyses showed clear early separation of survival curves for 3‐month mRS >2 versus 0 to 2, which persisted at 5 years (Figure 3). By 5 years, the probability of survival was similar for patients with 3‐month mRS scores of 3 and 4, with an additional drop observed for mRS 5.

**Figure 4:**
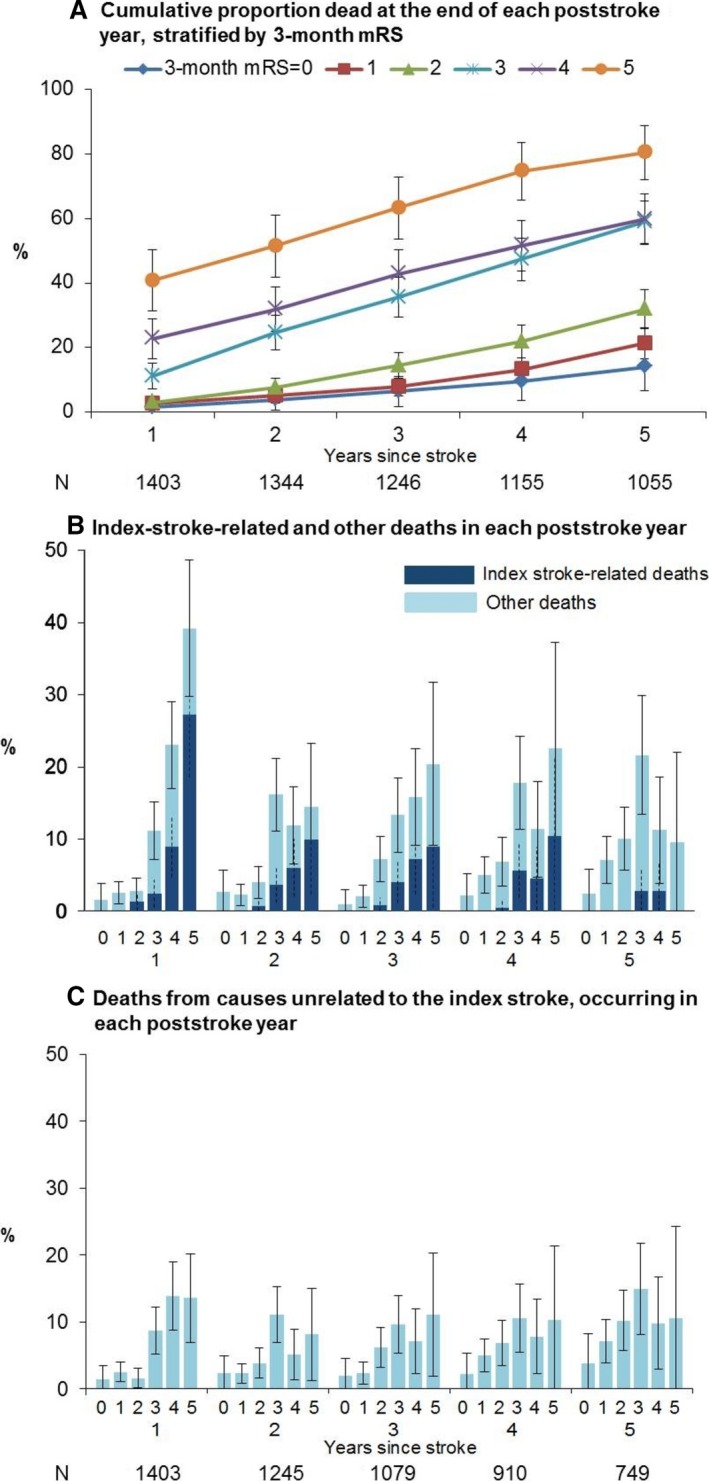
Proportions of 3‐month ischemic stroke survivors, categorized according to pre‐3‐month mRS, representing (A) cumulative deaths at the end of each poststroke year, (B) new deaths at the end of each poststroke year from causes related and unrelated to the index stroke, and (C) only from non‐stroke‐related causes, for poststroke years 1 through 5. Only patients with known follow‐up status (dead/alive) for a given year are included in the denominator for that year in (A); (B and C) further restrict the denominator to those who were alive at the start of each year. These numbers are shown below (A and C) respectively. Bars (solid for all‐cause, dashed for stroke‐related deaths) represent 95% confidence intervals. mRS, indicates modified Rankin Scale.

On examining the time course of 1‐ to 5‐year deaths, categorized according to 3‐month mRS, the cumulative death rate during each year remained higher for mRS >2 than for 0 to 2, with a step change from mRS 2 to 3 (Figure 4A). However, mortality differences between mRS 0 to 2 and >2—and among mRS categories 3, 4, and 5—were most pronounced in year 1. Nonstroke deaths occurred less frequently in year 1 in the lower mRS categories but were less clearly distributed in subsequent years (Figure 4B and 4C). The rising burden of nonstroke deaths in the lower disability categories accounted for some erosion of mortality differences among mRS categories over time.

On age‐ and sex‐adjusted Cox regressions, 3‐month mRS >2 strongly predicted 1‐, 2‐, 3‐, 4‐, and 5‐year mortality (Tables S2 and S3), although the adjusted hazard ratio for mRS >2 versus 0 to 2 from 3‐months to 1‐year (6.67, 95%CI 4.16 to 10.69) was greater than that for 3 months to 5 years (2.93, 95%CI 2.38 to 3.60, *P*<0.0001). On using the full range of the mRS in the age‐ and sex‐adjusted Cox models for 1‐ and 5‐year mortality, the mRS gained predictive value with each increment of the scale (Table S4).

### Disability and Combined Disability/Death Outcomes

Among 5‐year survivors, 3‐month mRS also predicted disability at 5 years (Figure 2C). In particular, there was a step change between mRS 2 and 3, with 88/125 (70.4%) of those with 3‐month mRS of 3 remaining disabled at 5 years versus 29/231 (12.6%) of those with 3‐month mRS of 2 (*P*<0.0001). However, the mRS did often change between 3 months and 1 year (Table 4), with recovery by 1 or more grades observed in 317/1266 (25.0%) patients with 3‐month mRS≥1. Recovery to functional independence (ie, an improvement to mRS 0 to 2) by 1 year occurred in 58 (23.1%) patients with 3‐month mRS of 3 versus 7 (3.9%) of those with 3‐month mRS of 4. Although all survivors with a 3‐month mRS of 5 remained disabled at 1 year, 16 (15.5%) showed an improvement by 1 or 2 grades. Accrual of new disability (mRS >2) at 1 year occurred in 75 (8.6%) patients who had a 3‐month mRS≤2. Recurrent strokes or other vascular events accounted for 38 (50.7%) of these deteriorations.

**Table 4:** mRS Changes Between 3 Months and 1 Year for All 3‐Month Stroke Survivors

	mRS 1 Y After Index Stroke							Improvement in mRS^a^ (%)	Total
	0	1	2	3	4	5	Death (%)		
3‐mo mRS									
0	63	61	9	2	0	0	2 (1.5)	N/A	137
1	66	252	76	18	4	0	11 (2.6)	66 (15.5)	427
2	22	104	119	42	9	0	9 (3.0)	126 (41.3)	305
3	0	13	45	128	33	4	28 (11.2)	58 (23.1)	251
4	0	0	7	44	77	11	41 (22.8)	51 (28.3)	180
5	0	0	0	1	15	45	42 (40.8)	16 (15.5)	103
Total	151	430	256	235	138	60	133 (9.5)	317 (25.0)	1403

mRS indicates modified Rankin Scale.

a Improvement refers to the 1‐year mRS score being 1 or more grades lower than the 3‐month mRS (3‐month mRS missing in 22 cases). *P*<0.0001. The denominator in each case is the row total, except for “317 (25.0),” where the denominator was 1266 (excluding the 137 patients with 3‐month mRS 0 who could not improve any further).

Change in mRS was less frequent between 1 and 5 years (Table 5). Whereas 91/858 (10.6%) patients with 1‐year mRS≥1 showed an improvement in the mRS by 1 or more grades, indicating some late recovery, only 13/353 (3.7%) survivors with 1‐year mRS of 3 or 4 were independent at 5 years. Similarly, new disability at 5 years occurred in only 10/573 (1.7%) patients with 1‐year mRS of 0 to 2.

**Table 5:** mRS Changes Between 1 and 5 Years for 3‐Month Stroke Survivors With Full 5 Years of Follow‐Up

	mRS 5 Y After Index Stroke							Improvement in mRS (%)	Total
	0	1	2	3	4	5	Death (%)		
1‐y mRS									
0	30	26	5	0	0	0	7 (10.3)	N/A	68
1	34	167	39	1	3	1	50 (16.9)	34 (11.5)	295
2	0	37	104	3	1	1	64 (30.5)	37 (17.6)	210
3	0	4	7	60	32	9	86 (43.4)	11 (5.6)	198
4	0	0	2	7	32	5	61 (57.0)	9 (8.4)	107
5	0	0	0	0	0	13	35 (72.9)	0	48
Total	64	234	157	71	68	29	303 (32.7)	91 (10.6)	926

*P*<0.0001. This table excludes 138 patients who died in poststroke year 1 and 348 patients who had not yet attained 5 years of follow‐up (censored cases). It includes 2 patients not included in Tables 4 and S5, for whom 1‐ and 5‐year mRS scores were available but for whom we had been unable to assess mRS at or before 3 months.

A step change from mRS 2 to 3 was also evident on grouping the composite outcome of 5‐year disability/death by 3‐month mRS (Figure 2D; overall mRS changes between 3 months and 5 years are shown in Table S5). A 3‐month mRS>2 strongly predicted 5‐year disability/death in age‐ and sex‐adjusted logistic regressions (Table S2); 723 (83.2%) patients in the overall cohort with 3‐month mRS of 0 to 2 were alive (or censored) at 5 years, of whom 47 (6.5%) were disabled, whereas 246 (46.1%) of those with 3‐month mRS>2 were alive/censored at 5 years, of whom 202 (82.1%) remained disabled (adjusted odds ratio for 5‐year disability/death for mRS>2 versus 0 to 2 = 34.15, 95%CI 23.57 to 49.48, *P*<0.0001). The models were all strong (c‐statistic≥0.80). Similar findings were observed after excluding patients with premorbid disability (Table S6).

### Subgroup Analyses

In addition to having more severe strokes (higher National Institutes of Health Stroke Scale), by definition, patients in the treatable major stroke group were generally older and more likely to have premorbid disability and comorbidities than the nonhyperacute/minor stroke group (Table S1). Similar differences were seen on comparing AF‐related and nonlacunar stroke patients versus non‐AF‐related and lacunar stroke patients, respectively. On adjusting comparisons for age and sex, most of these differences remained significant.

Although the lacunar stroke subgroup had lower mortality and fewer patients with mRS>2, similar trends were generally observed across the subgroups as in the overall cohort, with 3‐month mRS strongly predicting 1‐ and 5‐year mortality (Figure S1A and S1B; 1‐ and 5‐year mortality outcomes for subgroups displayed individually in Figures S2 and S3, Tables S2 and S3). Kaplan‐Meier analyses in the subgroups also showed clear early separation of survival curves for 3‐month mRS>2 versus 0 to 2, persisting at 5 years (Figure 3B through 3D and Figure S4). A step change from mRS 2 to 3 was also evident on grouping the outcomes of 5‐year disability and disability/death by 3‐month mRS (Figures S1C, S1D, S5, and S6, Table S6).

## Discussion

The time course of disability and cause‐specific mortality in the first year after stroke in our cohort demonstrates both late recovery from stroke‐related disability and magnification of mortality differences among mRS categories, driven by index‐stroke‐related deaths. Disability and mortality differences persist at 5 years despite some erosion by recurrent events and non‐stroke‐related deaths, with 3‐month mRS being a strong independent predictor of long‐term disability and mortality. After adjustment for age and sex, and on excluding patients with premorbid disability, we observed similar trajectories in long‐term death and disability in various clinical subgroups once categorized according to 3‐month mRS. These findings have implications for follow‐up and interpretation of stroke trials.

First, our results reaffirm the use of the 3‐month mRS as a pragmatic outcome measure in acute stroke trials. Our finding that 3‐month mRS score predicts 5‐year mortality, suggesting that reduction of early disability might reduce long‐term mortality, is in agreement with 4 prior studies that examined early stroke disability and long‐term mortality.^9^, ^10^, ^11^, ^13^ Our study adds a robust population‐based design, high rates of ascertainment of all incident strokes, assessment of causes of late poststroke death, completeness of follow‐up (1.5% missing), and replication of findings in important subgroups. Despite an elderly study population (mean age 73 years), we have further demonstrated that patients attaining low levels of poststroke disability (mRS 0 to 2) can generally be expected not only to survive longer but also to sustain their independence up to 5 years. These findings also highlight the importance of measuring stroke functional outcome even in trials in stroke prevention.

Second, our long‐term disability results demonstrate that extending acute stroke trial follow‐up to 1 year would almost completely capture long‐term stroke‐related disability. Whereas late recovery appears to occur in about 1 in 4 patients between 3 months and 1 year poststroke—notwithstanding potential inter‐/intrarater variability in mRS assessment—rates of further recovery or worsening decline between 1 and 5 years. However, if disability assessment is done beyond 1‐year follow‐up, earlier treatment effects appear unlikely to be significantly eroded by non‐stroke‐related factors. Nevertheless, our results indicate that studies of stroke treatments beyond the acute phase, such as later rehabilitation or stem‐cell therapies,^29^ should control for the late recovery that can be expected from a substantial proportion of patients in the first year.

Third, our assessment of the causes of poststroke death demonstrates that although over half of post‐3‐month index‐stroke‐related deaths occurred after 1year, the 1‐year mRS did strongly predict these later deaths. This helps reassure us that those subsequent deaths were indeed likely related to enduring disability from the index stroke. Mortality associations with early stroke‐related disability are still evident at 5 years despite some erosion from non‐stroke‐related deaths. Consequently, although some might question the necessity of extending mortality follow‐up beyond 1 year, our findings support the conclusions from follow‐up of the IST‐3 trial that there is significant potential to demonstrate translation of early disability gains into additional reductions in long‐term mortality.^4^ Trials might limit face‐to‐face or telephone follow‐up of disability to 1 year, but using administrative methods to obtain mortality follow‐up to 2 years or beyond appears to be justified.

Our findings also have implications for health‐economic analyses. Cost‐effectiveness analyses of stroke therapies have either assumed a static post‐3‐month outcome for dependent patients without any added mortality from their disability^30^ or used hazard ratios generated from expert panels rather than real‐world data,^31^ which may have underestimated the long‐term mortality risk of stroke‐related disability.^32^ Accounting for the potential for a state change from disabled to nondisabled, particularly in the first year poststroke as seen in our study, could also add to the robustness of cost‐effectiveness models. Longer‐term costing and utility data would allow more accurate modeling of expected healthcare cost savings and quality‐adjusted life‐year gains from shifts in 3‐month mRS.

Athough our analysis has several strengths, including generalizability, with similar 5‐year mortality and disability to the 1‐ and 5‐year results reported by prior population‐based studies,^33^, ^34^, ^35^ there are some potential shortcomings. First, a randomized controlled trial would be required to prove a causal association between lowering early disability and reducing mortality. However, our findings imply that treatments promoting an early shift toward lower mRS scores could reduce both long‐term disability and mortality. Second, our analysis of the causes of poststroke death is limited by the practical difficulty of establishing reliably in all cases whether an observed death is causally related to disability from the index stroke, particularly beyond 1 year, and especially in relation to a more direct cause of death such as pneumonia or pulmonary embolism. Although we sought to minimize this limitation by using overlapping sources to ascertain as much information as possible about the circumstances of deaths, we did not formally assess inter‐ or intrarater reliability in the designation of index‐stroke‐related deaths. We also likely underestimated the number of index‐stroke‐related deaths by coding deaths from recurrent strokes as being unrelated to the index stroke. Third, it could also be argued that because we defined long‐term disability as mRS>2, this made it more likely for us to find a step change from 3‐month mRS 2 to 3 for the 5‐year disability outcome. However, we found the same step change for 5‐year mortality. Fourth, our results may not be entirely generalizable to a clinical trial population owing to differences between patients in our population‐based cohort and those who meet enrollment criteria for trials.

In conclusion, our study highlights the predictive value of the short‐term mRS for long‐term clinical outcomes after stroke, with implications for trial follow‐up and outcome assessment more generally, including cost‐effectiveness analyses. Further work is required to document the time course of long‐term quality‐adjusted life expectancy and healthcare costs in relation to early mRS scores.

## Sources of Funding

The Oxford Vascular Study has been funded by Wellcome Trust, Wolfson Foundation, and the NIHR Oxford Biomedical Research Centre. Rothwell is in receipt of an NIHR Senior Investigator Award and a Wellcome Trust Senior Investigator Award. Ganesh is funded by the Rhodes Trust. We acknowledge the support of the Acute Vascular Imaging Centre, John Radcliffe Hospital, Oxford, UK.

## Disclosures

None.

## Supporting information


**Data S1.** Supplemental methods.
**Table S1.** Patient Sample and Characteristics for All 3‐Month Survivors of Ischemic Stroke and Subgroups of Interest
**Table S2.** Impact of 3‐Month mRS (>2 Versus 0‐2) on Disability/Death at 5 Years (Logistic Regression) and 5‐Year Mortality (Cox Regression) for All 3‐Month Ischemic Stroke Survivors and Subgroups, Adjusted for Age and Sex
**Table S3.** Impact of Dichotomized 3‐Month mRS (>2 Versus 0‐2) on Mortality for Poststroke Years 1 to 4 for 3‐Month Stroke Survivors—Controlling for Age and Sex—Including Treatable Major Strokes and Related Subgroups
**Table S4.** Impact of the 3‐Month mRS (Using the Full Range of Scores From 0 to 5) on 1‐ and 5‐Year Mortality for 3‐Month Stroke Survivors—Controlling for Age and Sex
**Table S5.** mRS Changes Between 3 Months and 5 Years (or 1 Year if Censored) for 3‐Month Stroke Survivors
**Table S6.** Impact of Dichotomized 3‐Month mRS (0‐2 Versus >2) on Disability or Death at 5 Years for 3‐Month Stroke Survivors, for the Overall Cohort and Relevant Subgroups—Controlling for Age and Sex and Excluding Patients With Premorbid Disability
**Figure S1.** Proportions of 3‐month ischemic stroke survivors in subgroups, stratified by 3‐month mRS, who were (A) dead at 1 year; (B) dead at 5 years; (C) disabled (mRS >2) at 5 years, only including those alive at 5 years; and (D) dead/disabled at 5 years.
**Figure S2.** Proportions of 3‐month survivors of ischemic stroke for key subgroups, stratified by 3‐month mRS, who were dead at 1 year (2 years for the lacunar subgroup, as there was only 1 death in the first year). Bars represent 95% confidence intervals.
**Figure S3.** Proportions of 3‐month survivors of ischemic stroke for key subgroups, stratified by pre‐3‐month mRS, who were dead at 5 years. Bars represent 95% confidence intervals.
**Figure S4.** Kaplan‐Meier survival curves after index stroke for 3‐month survivors (mRS 0‐2 versuss 3, 4, 5), for (A) nonhyperacute/minor strokes, (B) non‐AF‐related strokes, and (C) nonlacunar stroke subgroups. *P* values obtained from the log‐rank test for each group are displayed.
**Figure S5.** Proportions of 3‐month survivors of ischemic stroke for key subgroups, stratified by pre‐3‐month mRS, who were disabled (mRS>2) at 5 years, only including those alive at 5 years in the total. Bars represent 95% confidence intervals.
**Figure S6.** Proportions of 3‐month survivors of ischemic stroke for key subgroups, stratified by pre‐3‐month mRS, who were dead or disabled at 5 years. Bars represent 95% confidence intervals.
