# Supplementary material for: Time Course of Evolution of Disability and Cause‐Specific Mortality After Ischemic Stroke: Implications for Trial Design
Source: J Am Heart Assoc. 2017 Jun 11;6(6):e005788. doi: 10.1161/JAHA.117.005788 (PMC5669183; doi:10.1161/JAHA.117.005788)
Supplement: Supplementary file 1 — Data S1. Supplemental methods. Table S1. Patient Sample and Characteristics for All 3‐Month Survivors of Ischemic Stroke and Subgroups of Interest Table S2. Impact of 3‐Month mRS (>2 Versus 0‐2) on Disability/Death at 5 Years (Logistic Regression) and 5‐Year Mortality (Cox Regression) for All 3‐Month Ischemic Stroke Survivors and Subgroups, Adjusted for Age and Sex Table S3. Impact of Dichotomized 3‐Month mRS (>2 Versus 0‐2) on Mortality for Poststroke Years 1 to 4 for 3‐Month Stroke Survivors—Controlling for Age and Sex—Including Treatable Major Strokes and Related Subgroups Table S4. Impact of the 3‐Month mRS (Using the Full Range of Scores From 0 to 5) on 1‐ and 5‐Year Mortality for 3‐Month Stroke Survivors—Controlling for Age and Sex Table S5. mRS Changes Between 3 Months and 5 Years (or 1 Year if Censored) for 3‐Month Stroke Survivors Table S6. Impact of Dichotomized 3‐Month mRS (0‐2 Versus >2) on Disability or Death at 5 Years for 3‐Month Stroke Survivors, for the Overall Cohort and Relevant Subgroups—Controlling for Age and Sex and Excluding Patients With Premorbid Disability Figure S1. Proportions of 3‐month ischemic stroke survivors in subgroups, stratified by 3‐month mRS, who were (A) dead at 1 year; (B) dead at 5 years; (C) disabled (mRS >2) at 5 years, only including those alive at 5 years; and (D) dead/disabled at 5 years. Figure S2. Proportions of 3‐month survivors of ischemic stroke for key subgroups, stratified by 3‐month mRS, who were dead at 1 year (2 years for the lacunar subgroup, as there was only 1 death in the first year). Bars represent 95% confidence intervals. Figure S3. Proportions of 3‐month survivors of ischemic stroke for key subgroups, stratified by pre‐3‐month mRS, who were dead at 5 years. Bars represent 95% confidence intervals. Figure S4. Kaplan‐Meier survival curves after index stroke for 3‐month survivors (mRS 0‐2 versuss 3, 4, 5), for (A) nonhyperacute/minor strokes, (B) non‐AF‐related strokes, and (C) nonlacunar stroke subgroup [file JAH3-6-e005788-s001.pdf]

# SUPPLEMENTAL MATERIAL

**Data S1.**

## Supplemental Methods

### Assessment of Cause of Death

Deaths and causes of death were ascertained through monthly visits to the coroner's office, review of all death certificates in the study's general practices, practice-specific listings of all ICD-10 (International Classification of Diseases) death codes registered centrally, and daily identification via bereavement officers of patients who were dead on arrival at the hospital or who died soon after. If a patient died before assessment, we obtained an eyewitness account of the clinical event and reviewed any relevant records. If death took place outside the hospital or before investigation, the autopsy result was reviewed. Clinical details were sought from primary-care physicians or other clinicians on all deaths occurring in this cohort of ischaemic stroke survivors.

A list of all deaths occurring in Oxfordshire with a vascular ICD-10 code in any position on the death certificate is generated by the local Department of Public Health. These anonymised data are provided on a three-monthly basis. After a patient dies their GP notes are sent to central storage at the Thames Valley Health Authority headquarters. These notes were used to collect further past medical history, supplement missing data, and ensure accurate diagnosis of cause of death.

For the purposes of this analysis, deaths were coded as related to: (1) a recurrent ischaemic or haemorrhagic stroke, either acutely (<30 days) or non-acutely as a result of further stroke-related disability (ICD I607, I609, I610, I619, I620, I634, I635, I639, I64X, I678, I679, I694, I698); (2) a cardiac cause if the primary cause of death was deemed to be related to heart disease, including acute coronary syndromes (ICD I219, I251) and congestive heart failure; (3) pneumonia or other chest infection; (4) sepsis or other severe infection including meningitis and endocarditis; (5) dementia; (6) pulmonary embolism and/or deep-vein thrombosis (PE/DVT); (7) peripheral vascular disease (ICD I709, I710, I711, I713, I714, I718, I719 , I728, I739); (8) cancer; (9) renal failure from acute or chronic kidney disease; (10) gastro-intestinal bleeding; (11) fall or similar trauma, including traumatic subdural haematoma or traumatic subarachnoid haemorrhage; (12) chronic obstructive pulmonary disease or other chronic lung disease;  or (13) other cause, if the death did not fall into any of the aforementioned categories.

**Table S1. Patient sample and characteristics for all 3-month survivors of ischaemic stroke and subgroups of interest**

| | **Treatable major stroke patients** (n=183) | **Non-hyperacute/ minor stroke patients** (n=1,243) | Age- and sex-adjusted P | **AF-related stroke patients** (n=329) | **Non-AF-related stroke patients** (n=1,096) | Age- and sex-adjusted P | **Lacunar stroke patients** (n=234) | **Non-lacunar stroke patients** (n=1,191) | Age- and sex-adjusted P |
|---|---|---|---|---|---|---|---|---|---|
| **Age, mean (S.D.)** | 75.3 (11.9) | 72.9 (12.8) | | 78.3 (9.8) | 71.7 (13.1) | | 69.9 (12.0) | 73.8 (12.8) | |
| **Sex – male (%)** | 90 (49.2) | 663 (53.3) | | 168 (51.1) | 585 (53.4) | | 143 (61.1) | 610 (51.2) | |
| **Previous history(%):** | | | | | | | | | |
| MI | 31 (16.9) | 146 (11.8) | **0.060*** | 61 (18.5) | 116 (10.6) | **0.001*** | 13 (5.6) | 164 (13.8) | **0.002*** |
| Angina | 30 (16.4) | 209 (16.8) | 0.71 | 79 (24.0) | 160 (14.6) | **0.007*** | 32 (13.7) | 207 (17.4) | 0.46 |
| Atrial Fibrillation | 54 (29.5) | 206 (16.6) | **<0.001*** | 257 (78.1) | 3 (0.3) | **<0.001*** | 2 (0.9) | 258 (21.7) | **<0.001*** |
| Hypertension | 120 (65.6) | 769 (61.9) | 0.63 | 231 (70.2) | 658 (60.0) | 0.10 | 145 (62.0) | 744 (62.5) | 0.87 |
| Dyslipidemia | 60 (32.8) | 409 (32.9) | 0.98 | 118 (35.9) | 351 (32.0) | 0.11 | 72 (30.8) | 397 (33.3) | 0.45 |
| Diabetes | 23 (12.6) | 183 (14.7) | 0.48 | 50 (15.2) | 155 (14.1) | 0.64 | 42 (17.9) | 163 (13.7) | 0.10 |
| PVD | 15 (8.2) | 93 (7.5) | 0.75 | 31 (9.4) | 77 (7.0) | 0.21 | 13 (5.6) | 95 (8.0) | 0.31 |
| Stroke | 19 (10.4) | 139 (11.2) | 0.54 | 53 (16.1) | 105 (9.6) | **0.03*** | 22 (9.4) | 136 (11.4) | 0.66 |
| TIA | 26 (14.2) | 179 (14.4) | 0.74 | 67 (20.4) | 138 (12.6) | **0.02*** | 29 (12.4) | 176 (13.8) | 0.75 |
| Smoking | 108 (59.0) | 729 (58.7) | 0.57 | 186 (56.5) | 650 (59.3) | 0.98 | 147 (62.8) | 689 (57.9) | 0.70 |
| Cancer | 37 (20.2) | 184 (14.8) | 0.15 | 61 (18.5) | 160 (14.6) | 0.90 | 32 (13.7) | 189 (15.9) | 0.61 |
| Prior disability (mRS ≥3) | 38 (20.8) | 206 (16.6) | 0.62 | 76 (23.2) | 168 (15.4) | 0.45 | 17 (7.3) | 227 (19.1) | **0.003*** |
| **NIHSS**, median (IQR) | 10 (7-15) | 2 (0-3) | **<0.001*** | 3 (1-8) | 2 (0-4) | **<0.001*** | 2 (1-3) | 2 (1-5) | **<0.001*** |

Significant differences ($p<0.05$) between groups are indicated by an asterisk (*). For each subgroup pair (Treatable major strokes versus non-hyperacute/minor strokes, AF-related versus non-AF-related strokes, and lacunar versus non-lacunar strokes), age was compared using an independent samples T-test, while median and distribution of NIHSS were compared using independent-samples Median and Mann-Whitney U Tests respectively. The remaining dichotomous variables were compared using the Chi-squared test. Abbreviations: AF – Atrial Fibrillation, S.D. – Standard deviation, MI – Myocardial Infarction, PVD – Peripheral Vascular Disease, TIA – Transient Ischaemic Attack, mRS – modified Rankin scale, NIHSS – National Institutes of Health Stroke Scale, IQR – Inter-quartile range.

**Table S2. Impact of 3-month mRS (>2 versus 0-2) on disability/death at 5-years (logistic regression) and 5-year mortality (Cox regression) for all 3-month ischaemic stroke survivors and subgroups, adjusted for age and sex**

| | mRS >2 versus 0-2 | | Age | | Male | | N | c-statistic |
|---|---|---|---|---|---|---|---|---|
| | aOR/HR(95%CI) | p>\|z\| | aOR/HR | p>\|z\| | aOR/HR | p>\|z\| | | |
| **All stroke patients** | | | | | | | | |
| aOR (death/ disability) | 34.15 (23.57-49.48) | <0.0001* | 1.08 (1.06-1.09) | <0.0001* | 0.81(0.60-1.10) | 0.180 | 1403 | 0.90 |
| aHR(death) | 2.93 (2.38-3.60) | <0.0001* | 1.08 (1.07-1.09) | <0.0001* | 1.25(1.03-1.52) | 0.022* | | |
| **Treatable major strokes** | | | | | | | | |
| aOR | 41.65(13.31-130.36) | <0.0001* | 1.08 (1.03-1.12) | <0.0001* | 2.08(0.68-6.37) | 0.198 | 181 | 0.91 |
| aHR | 2.40 (1.19-4.86) | 0.015* | 1.10 (1.07-1.14) | <0.0001* | 1.83(1.13-2.95) | 0.015* | | |
| **Non-hyperacute/minor strokes** | | | | | | | | |
| aOR | 29.00 (19.37-43.42) | <0.0001* | 1.08 (1.06-1.10) | <0.0001* | 0.74 (0.54-1.02) | 0.068 | 1222 | 0.89 |
| aHR | 2.92 (2.33-3.66) | <0.0001* | 1.07 (1.06-1.09) | <0.0001* | 1.19 (0.96-1.47) | 0.119 | | |
| **AF-related strokes** | | | | | | | | |
| aOR | 27.64 (13.09-58.35) | <0.0001* | 1.08 (1.04-1.12) | <0.0001* | 1.01(0.53-1.90) | 0.988 | 320 | 0.89 |
| aHR | 2.65 (1.81-3.87) | <0.0001* | 1.07 (1.05-1.10) | <0.0001* | 1.44(1.04-1.99) | 0.030* | | |
| **Non-AF-related strokes** | | | | | | | | |
| aOR | 35.19 (22.94-53.99) | <0.0001* | 1.07 (1.06-1.09) | <0.0001* | 0.76(0.54-1.07) | 0.117 | 1083 | 0.90 |
| aHR | 2.92 (2.28-3.75) | <0.0001* | 1.08 (1.06-1.09) | <0.0001* | 1.15(0.90-1.47) | 0.255 | | |
| **Lacunar strokes** | | | | | | | | |
| aOR | 8.97 (4.13-19.49) | <0.0001* | 1.09 (1.04-1.13) | <0.0001* | 0.72(0.35-1.47) | 0.369 | 234 | 0.85 |
| aHR | 1.18 (0.59-2.35) | 0.633 | 1.11 (1.06-1.16) | <0.0001* | 1.32(0.68-2.56) | 0.417 | | |
| **Non-lacunar strokes** | | | | | | | | |
| Aor | 45.89 (29.54-71.30) | <0.0001* | 1.08 (1.06-1.10) | <0.0001* | 0.86(0.61-1.20) | 0.377 | 1169 | 0.91 |
| Ahr | 3.16 (2.53-3.94) | <0.0001* | 1.07 (1.06-1.09) | <0.0001* | 1.27(1.04-1.56) | 0.021* | | |

p>|$X^2$| was <0.0001 for all the models. Significant p-values (<0.05) are indicated by an asterisk(*).

Abbreviations: aOR – adjusted odds ratio from logistic regression for 5-year disability/death, aHR – adjusted hazard ratio from Cox regression for 5-year mortality

**Table S3. Impact of dichotomized 3-month mRS (>2 vs 0-2) on mortality for post-stroke years 1 to 4 for 3-month stroke survivors – controlling for age and sex – including treatable major strokes and related subgroups.**

| | mRS >2 versus 0-2 | | Age | | Male | | Total (n) |
|---|---|---|---|---|---|---|---|
| | Adj. HR (95% CI) | p>\|z\| | Adj. HR (95% CI) | p>\|z\| | Adj. HR (95% CI) | p>\|z\| | |
| **All stroke patients** | | | | | | | |
| Year 1 | 6.67 (4.16-10.69) | <0.0001* | 1.06 (1.04-1.08) | <0.0001* | 1.55 (1.09-2.20) | 0.014* | 1403 |
| Year 2 | 4.80 (3.43-6.71) | <0.0001* | 1.07 (1.05-1.08) | <0.0001* | 1.32 (1.01-1.74) | 0.046* | |
| Year 3 | 3.86 (2.94-5.08) | <0.0001* | 1.07 (1.06-1.09) | <0.0001* | 1.17 (0.93-1.49) | 0.186 | |
| Year 4 | 3.34 (2.64-4.22) | <0.0001* | 1.08 (1.07-1.09) | <0.0001* | 1.26 (1.02-1.56) | 0.032* | |
| **Treatable major strokes** | | | | | | | |
| Year 1 | 9.61 (1.31-70.43) | 0.026* | 1.09 (1.04-1.14) | <0.0001* | 1.75 (0.86-3.53) | 0.120 | 181 |
| Year 2 | 14.2 (1.95-103.2) | 0.009* | 1.10 (1.05-1.14) | <0.0001* | 2.06 (1.12-3.78) | 0.020* | |
| Year 3 | 2.51 (1.14-5.55) | 0.023* | 1.11 (1.07-1.15) | <0.0001* | 1.83 (1.07-3.12) | 0.027* | |
| Year 4 | 2.42 (1.15-5.09) | 0.020* | 1.11 (1.07-1.15) | <0.0001* | 1.70 (1.02-2.81) | 0.040* | |
| **Non-hyperacute/minor strokes** | | | | | | | |
| Year 1 | 5.97 (3.59-9.93) | <0.0001* | 1.05 (1.03-1.08) | <0.0001* | 1.59 (1.06-2.40) | 0.027* | 1222 |
| Year 2 | 4.34 (3.03-6.21) | <0.0001* | 1.06 (1.04-1.08) | <0.0001* | 1.22 (0.89-1.67) | 0.210 | |
| Year 3 | 3.87 (2.87-5.23) | <0.0001* | 1.07 (1.05-1.09) | <0.0001* | 1.09 (0.83-1.42) | 0.544 | |
| Year 4 | 3.35 (2.60-4.32) | <0.0001* | 1.08 (1.06-1.09) | <0.0001* | 1.21 (0.96-1.54) | 0.107 | |
| **AF-related strokes** | | | | | | | |
| Year 1 | 10.48 (3.20-34.3) | <0.0001* | 1.06 (1.02-1.10) | 0.006* | 1.55 (0.86-2.81) | 0.148 | 320 |
| Year 2 | 5.20 (2.64-10.25) | <0.0001* | 1.07 (1.04-1.11) | <0.0001* | 1.56 (0.99-2.47) | 0.056 | |
| Year 3 | 3.85 (2.29-6.49) | <0.0001* | 1.07 (1.04-1.10) | <0.0001* | 1.37 (0.92-2.03) | 0.117 | |
| Year 4 | 3.21 (2.08-4.96) | <0.0001* | 1.07 (1.05-1.10) | <0.0001* | 1.40 (0.98-2.00) | 0.061 | |
| **Non-AF-related strokes** | | | | | | | |
| Year 1 | 5.94 (3.51-10.07) | <0.0001* | 1.06 (1.03-1.08) | <0.0001* | 1.54 (1.00-2.38) | 0.051 | 1083 |
| Year 2 | 4.55 (3.07-6.73) | <0.0001* | 1.06 (1.04-1.08) | <0.0001* | 1.19 (0.84-1.68) | 0.333 | |
| Year 3 | 3.74 (2.70-5.16) | <0.0001* | 1.07 (1.06-1.09) | <0.0001* | 1.06 (0.79-1.44) | 0.681 | |
| Year 4 | 3.27 (2.47-4.32) | <0.0001* | 1.08 (1.06-1.10) | <0.0001* | 1.18 (0.90-1.53) | 0.227 | |
| **Lacunar strokes** | | | | | | | |
| Year 1 | Only 1 death, so N/A | | | | | | 234 |
| Year 2 | 1.99 (0.52-9.55) | 0.389 | 1.15 (1.03-1.28) | 0.011* | 2.31(0.52-10.21) | 0.269 | |
| Year 3 | 1.68 (0.68-4.19) | 0.262 | 1.12 (1.06-1.20) | <0.0001* | 1.00 (0.41-2.43) | 0.996 | |
| Year 4 | 1.50 (0.67-3.34) | 0.324 | 1.12 (1.06-1.19) | <0.0001* | 1.14 (0.52-2.49) | 0.739 | |
| **Non-lacunar strokes** | | | | | | | |
| Year 1 | 6.66 (4.13-10.75) | <0.0001* | 1.05 (1.03-1.08) | <0.0001* | 1.57 (1.11-2.23) | 0.011* | 1169 |
| Year 2 | 4.78 (3.39-6.74) | <0.0001* | 1.06 (1.04-1.08) | <0.0001* | 1.33 (1.01-1.76) | 0.046* | |
| Year 3 | 4.06 (3.04-5.41) | <0.0001* | 1.07 (1.05-1.09) | <0.0001* | 1.21 (0.95-1.55) | 0.126 | |
| Year 4 | 3.51 (2.74-4.48) | <0.0001* | 1.07 (1.06-1.09) | <0.0001* | 1.29 (1.04-1.61) | 0.021* | |

p>$|X^2|$ was <0.0001 for each regression presented, except for Lacunar Strokes in Year 2, for which it was 0.0027.

**Table S4. Impact of the 3-month mRS (using the full range of scores from 0 to 5) on 1-year and 5-year mortality for 3-month stroke survivors – controlling for age and sex.**

| | 1-year Mortality | | 5-year Mortality | |
|---|---|---|---|---|
| | **aHR(95%CI)** | **p>\|z\|** | **aHR(95%CI)** | **p>\|z\|** |
| **3-month mRS** | 0=Reference | | 0=Reference | |
| **1** | 1.57 (0.35-7.08) | 0.560 | 1.36 (0.73-2.52) | 0.337 |
| **2** | 1.53 (0.33-7.12) | 0.587 | 1.89 (1.02-3.49) | 0.042* |
| **3** | 4.62 (1.09-19.67) | 0.038* | 3.53 (1.93-6.43) | <0.0001* |
| **4** | 11.15 (2.66-46.73) | 0.001* | 4.58 (2.49-8.43) | <0.0001* |
| **5** | 26.24 (6.27-109.76) | <0.0001* | 9.00 (4.84-16.74) | <0.0001* |
| **Age** | 1.07 (1.04-1.09) | <0.0001* | 1.08 (1.07-1.09) | <0.0001* |
| **Male** | 1.95 (1.36-2.80) | <0.0001* | 1.39 (1.14-1.69) | 0.001* |
| | $p>\|X^2\|$ | <0.0001* | $p>\|X^2\|$ | <0.0001* |
| | n | 1403 | n | 1403 |

## Table S5. mRS changes between 3 months and 5 years (or 1 year if censored) for 3-month stroke survivors

**All stroke patients**

| | mRS 5 years after index stroke | | | | | | | |
|---|---|---|---|---|---|---|---|---|
| | **0** | **1** | **2** | **3** | **4** | **5** | **Death (%)** | **Total** |
| **3-month mRS** | | | | | | | | |
| **0** | 55 | 60 | 10 | 0 | 0 | 0 | 12 (8.8) | 137 |
| **1** | 67 | 218 | 64 | 11 | 6 | 1 | 60 (14.1) | 427 |
| **2** | 25 | 84 | 93 | 19 | 8 | 2 | 74 (24.3) | 305 |
| **3** | 0 | 8 | 29 | 53 | 29 | 6 | 126 (50.2) | 251 |
| **4** | 0 | 0 | 7 | 23 | 47 | 11 | 92 (51.1) | 180 |
| **5** | 0 | 0 | 0 | 3 | 10 | 20 | 70 (68.0) | 103 |
| **Total** | 147 | 370 | 203 | 109 | 100 | 40 | 434 (30.9) | 1403 |

3-month mRS missing in 22 cases; n=348 censored; for all of them, mRS at 1 year was available and this was used instead. p<0.0001.

**Treatable major stroke patients**

| | mRS 5 years after index stroke | | | | | | | |
|---|---|---|---|---|---|---|---|---|
| | **0** | **1** | **2** | **3** | **4** | **5** | **Death (%)** | **Total** |
| **3-month mRS** | | | | | | | | |
| **0** | 2 | 1 | 0 | 0 | 0 | 0 | 0 | 3 |
| **1** | 2 | 8 | 0 | 1 | 1 | 0 | 2 (14.3) | 14 |
| **2** | 1 | 6 | 6 | 2 | 0 | 0 | 7 (31.8) | 22 |
| **3** | 0 | 1 | 5 | 11 | 2 | 1 | 18 (47.4) | 38 |
| **4** | 0 | 0 | 2 | 7 | 17 | 5 | 24 (43.6) | 55 |
| **5** | 0 | 0 | 0 | 1 | 6 | 10 | 32 (65.3) | 49 |
| **Total** | 5 | 16 | 13 | 22 | 26 | 16 | 83 (45.9) | 181 |

3-month mRS missing in 2 cases; n=36 censored; for all of them, mRS at 1 year was available and this was used instead. p<0.0001.

**Non-hyperacute/minor stroke patients**

| | mRS 5 years after index stroke | | | | | | | |
|---|---|---|---|---|---|---|---|---|
| | **0** | **1** | **2** | **3** | **4** | **5** | **Death (%)** | **Total** |
| **3-month mRS** | | | | | | | | |
| **0** | 53 | 59 | 10 | 0 | 0 | 0 | 12 (9.0) | 134 |
| **1** | 65 | 210 | 64 | 10 | 5 | 1 | 58 (14.0) | 413 |
| **2** | 24 | 78 | 87 | 17 | 8 | 2 | 67 (23.7) | 283 |
| **3** | 0 | 7 | 24 | 42 | 27 | 5 | 108 (50.7) | 213 |
| **4** | 0 | 0 | 5 | 16 | 30 | 6 | 68 (54.4) | 125 |
| **5** | 0 | 0 | 0 | 2 | 4 | 10 | 38 (70.4) | 54 |
| **Total** | 142 | 354 | 190 | 87 | 74 | 24 | 351 (28.7) | 1222 |

3-month mRS missing in 21 cases; n=312 censored; for all of them, mRS at 1 year was available and this was used instead. p<0.0001.

**AF-related stroke patients**

| | mRS 5 years after index stroke | | | | | | | |
|---|---|---|---|---|---|---|---|---|
| | **0** | **1** | **2** | **3** | **4** | **5** | **Death (%)** | **Total** |
| **3-month mRS** | | | | | | | | |
| **0** | 8 | 6 | 0 | 0 | 0 | 0 | 3 (17.7) | 17 |
| **1** | 7 | 33 | 11 | 3 | 0 | 0 | 15 (21.7) | 69 |
| **2** | 1 | 15 | 15 | 4 | 3 | 1 | 20 (33.9) | 59 |
| **3** | 0 | 1 | 8 | 13 | 12 | 1 | 50 (58.8) | 85 |
| **4** | 0 | 0 | 1 | 4 | 11 | 4 | 32 (61.5) | 52 |
| **5** | 0 | 0 | 0 | 0 | 3 | 6 | 29 (76.3) | 38 |
| **Total** | 16 | 55 | 35 | 24 | 29 | 12 | 149 (46.6) | 320 |

3-month mRS missing in 9 cases; n=59 censored; for all of them, mRS at 1 year was available and this was used instead. p<0.0001.

**Non-AF-related stroke patients**

| | mRS 5 years after index stroke | | | | | | | |
|---|---|---|---|---|---|---|---|---|
| | **0** | **1** | **2** | **3** | **4** | **5** | **Death (%)** | **Total** |
| **3-month mRS** | | | | | | | | |
| **0** | 47 | 54 | 10 | 0 | 0 | 0 | 9 (7.5) | 120 |
| **1** | 60 | 185 | 43 | 8 | 6 | 1 | 45 (12.6) | 358 |
| **2** | 24 | 69 | 78 | 15 | 5 | 1 | 54 (22.0) | 246 |
| **3** | 0 | 7 | 21 | 40 | 17 | 5 | 76 (45.8) | 166 |
| **4** | 0 | 0 | 6 | 19 | 36 | 7 | 60 (46.9) | 128 |
| **5** | 0 | 0 | 0 | 3 | 7 | 14 | 41 (63.1) | 65 |
| **Total** | 131 | 315 | 168 | 85 | 71 | 28 | 285 | 1083 |

3-month mRS missing in 13 cases; n=289 censored; for all of them, mRS at 1 year was available and this was used instead. p<0.0001.

**Lacunar stroke patients**

| | mRS 5 years after index stroke | | | | | | | |
|---|---|---|---|---|---|---|---|---|
| | **0** | **1** | **2** | **3** | **4** | **5** | **Death (%)** | **Total** |
| **3-month mRS** | | | | | | | | |
| **0** | 12 | 7 | 0 | 0 | 0 | 0 | 1 (5.0) | 20 |
| **1** | 19 | 48 | 15 | 1 | 2 | 1 | 10 (10.4) | 96 |
| **2** | 8 | 18 | 22 | 1 | 1 | 0 | 12 (19.4) | 62 |
| **3** | 0 | 3 | 11 | 10 | 0 | 1 | 12 (32.4) | 37 |
| **4** | 0 | 0 | 0 | 5 | 5 | 1 | 4 (26.7) | 15 |
| **5** | 0 | 0 | 0 | 1 | 1 | 2 | 0 | 4 |
| **Total** | 39 | 76 | 48 | 18 | 9 | 5 | 39 (16.7) | 234 |

3-month mRS available for all cases; n=55 censored; for all of them, mRS at 1 year was available and this was used instead. p<0.0001.

**Non-lacunar stroke patients**

| | mRS 5 years after index stroke | | | | | | | |
|---|---|---|---|---|---|---|---|---|
| | **0** | **1** | **2** | **3** | **4** | **5** | **Death (%)** | **Total** |
| **3-month mRS** | | | | | | | | |
| **0** | 43 | 53 | 10 | 0 | 0 | 0 | 11 (9.4) | 117 |
| **1** | 48 | 170 | 49 | 10 | 4 | 0 | 50 (15.1) | 331 |
| **2** | 17 | 66 | 71 | 18 | 7 | 2 | 62 (25.5) | 243 |
| **3** | 0 | 5 | 18 | 43 | 29 | 5 | 114 (53.3) | 214 |
| **4** | 0 | 0 | 7 | 18 | 42 | 10 | 88 (53.3) | 165 |
| **5** | 0 | 0 | 0 | 2 | 9 | 18 | 70 (70.7) | 99 |
| **Total** | 108 | 294 | 155 | 91 | 91 | 35 | 395 (33.8) | 1169 |

3-month mRS missing in 22 cases; n=293 censored; for all of them, mRS at 1 year was available and this was used instead. p<0.0001.

**Table S6. Impact of dichotomized 3-month mRS (0-2 versus >2) on disability or death at 5 years for 3-month stroke survivors , for the overall cohort and relevant subgroups – controlling for age and sex, and excluding patients with premorbid disability**

| | mRS >2 | | Age | | Male | | Total (n) | c-statistic |
|---|---|---|---|---|---|---|---|---|
| | Adj. OR (95% CI) | p>\|z\| | Adj. OR (95% CI) | p>\|z\| | Adj. OR (95% CI) | p>\|z\| | | |
| **All stroke patients** | 25.22 (16.85-37.74) | <0.0001* | 1.08 (1.06-1.09) | <0.0001* | 0.80 (0.59-1.10) | 0.172 | 1171 | 0.87 |
| **Treatable major strokes** | 31.49 (10.02-98.93) | <0.0001* | 1.07 (1.03-1.12) | 0.001* | 1.96 (0.54-6.00) | 0.237 | 145 | 0.90 |
| **Non-hyperacute/ minor strokes** | 19.80 (12.65-30.99) | <0.0001* | 1.08 (1.06-1.10) | <0.0001* | 0.74 (0.53-1.03) | 0.074 | 1026 | 0.85 |
| **AF-related strokes** | 31.86 (12.71-79.90) | <0.0001* | 1.08 (1.03-1.12) | <0.0001* | 1.00 (0.51-1.97) | 0.997 | 249 | 0.87 |
| **Non-AF-related strokes** | 22.52 (14.31-35.42) | <0.0001* | 1.07 (1.06-1.09) | <0.0001* | 0.74 (0.52-1.06) | 0.101 | 922 | 0.86 |
| **Lacunar strokes** | 6.42 (2.79-14.81) | <0.0001* | 1.09 (1.04-1.13) | <0.0001* | 0.64 (0.30-1.33) | 0.230 | 217 | 0.83 |
| **Non-lacunar strokes** | 35.52 (21.83-57.78) | <0.0001* | 1.08 (1.06-1.10) | <0.0001* | 0.87 (0.61-1.23) | 0.429 | 954 | 0.88 |

p>|$X^2$| was <0.0001 for each regression presented. Significant differences (p<0.05) between groups are indicated by an asterisk (*).

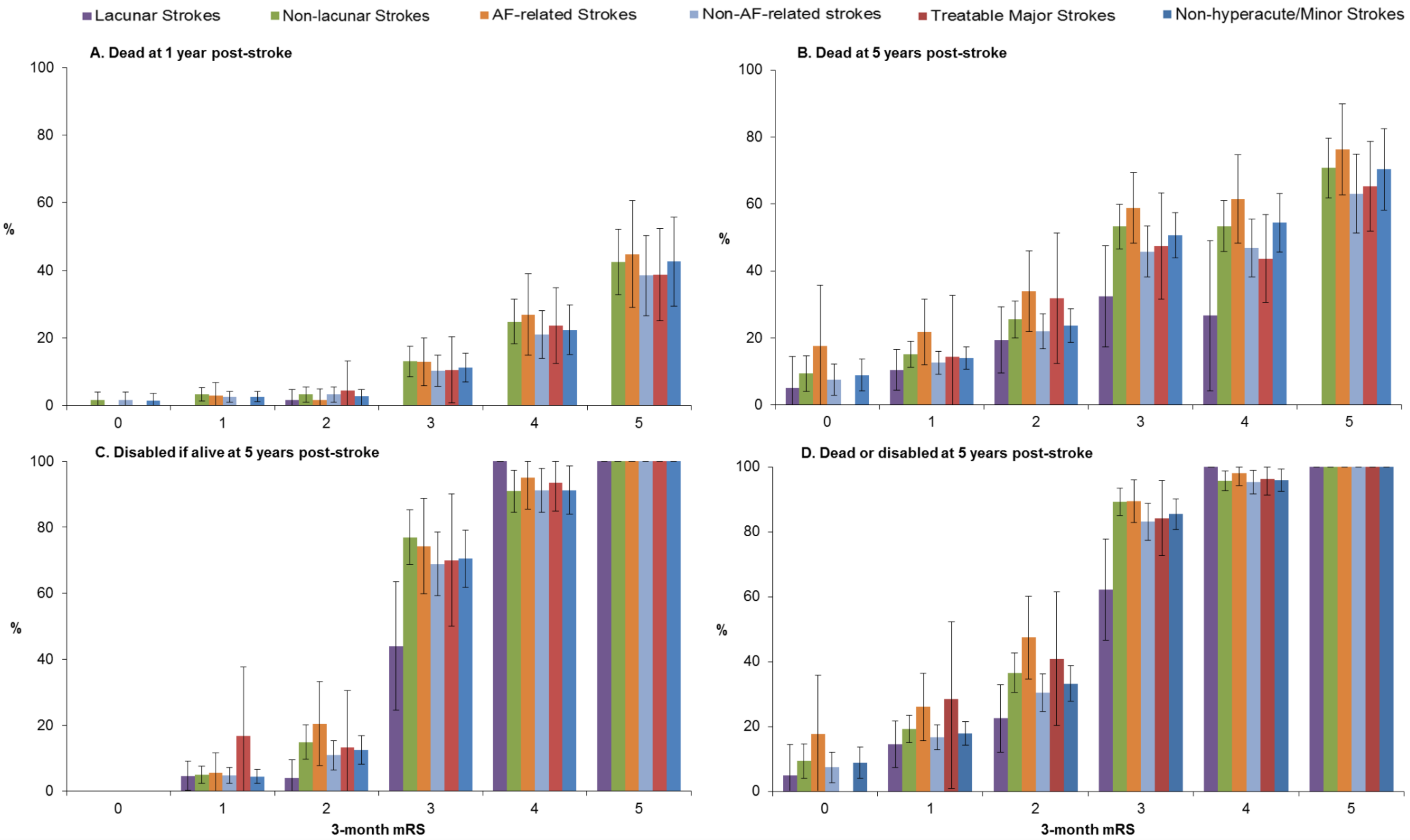


**Figure S1. Proportions of 3-month ischaemic stroke survivors in subgroups, stratified by 3-month mRS, who were: (A) dead at 1-year; (B) dead at 5-years; (C) disabled (mRS>2) at 5-years, only including those alive at 5-years; and (D) dead/disabled at 5-years.**

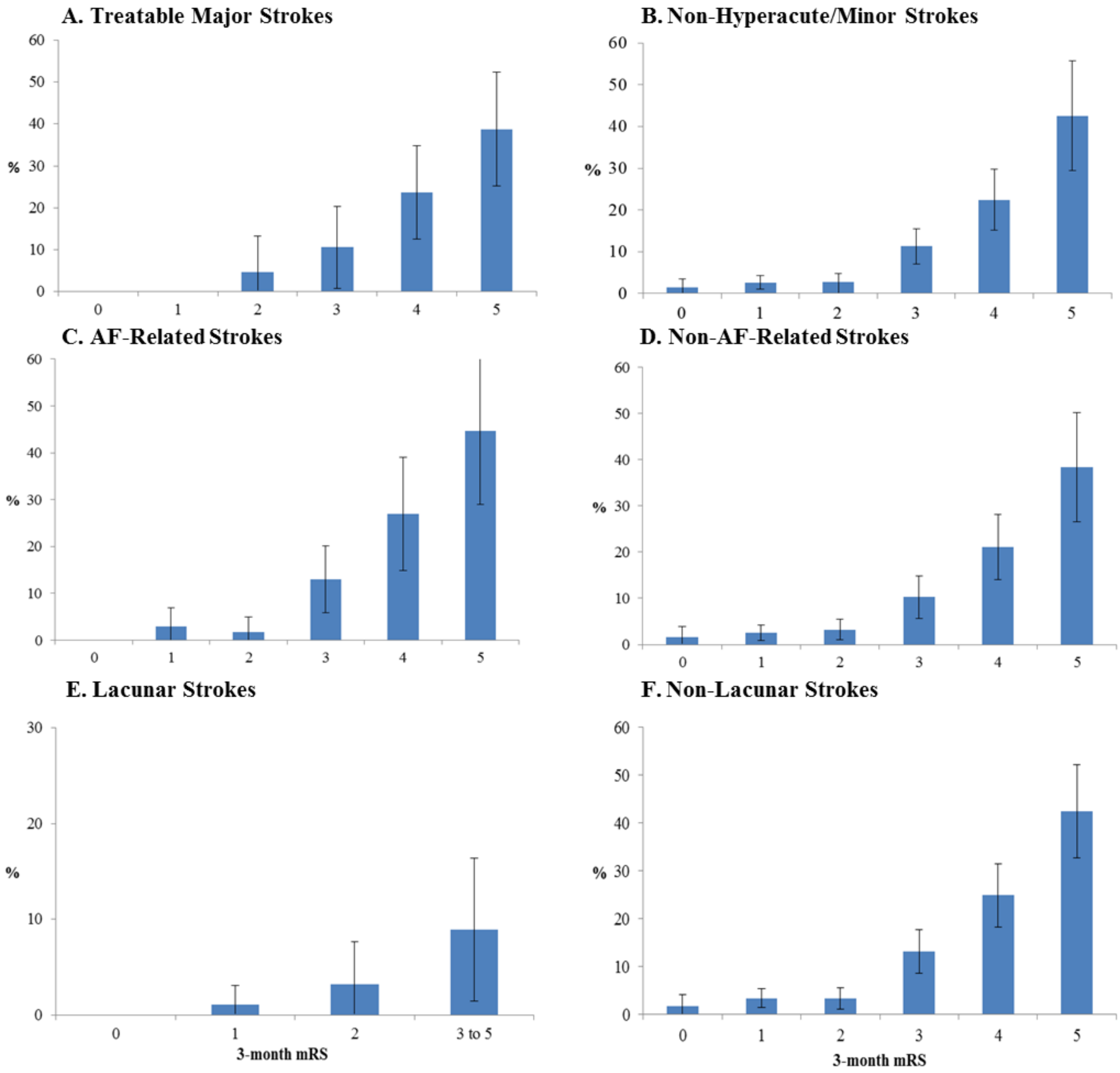


**Figure S2. Proportions of 3-month survivors of ischaemic stroke for key subgroups, stratified by 3-month mRS, who were dead at 1 year (2 years for the lacunar subgroup, as there was only 1 death in the first year). Bars represent 95% confidence intervals.**

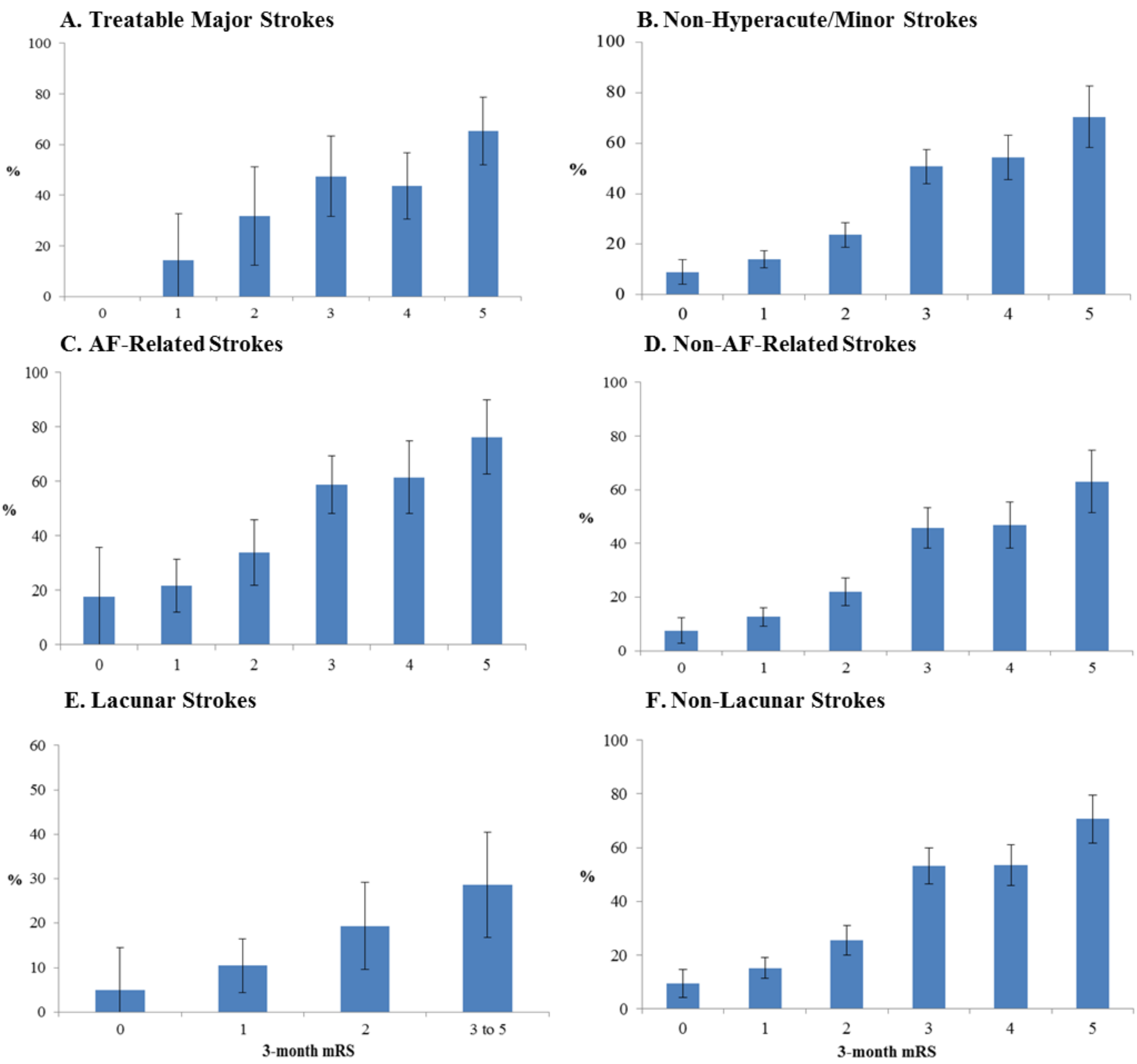


**Figure S3. Proportions of 3-month survivors of ischaemic stroke for key subgroups, stratified by pre-3-month mRS, who were dead at 5 years. Bars represent 95% confidence intervals.**

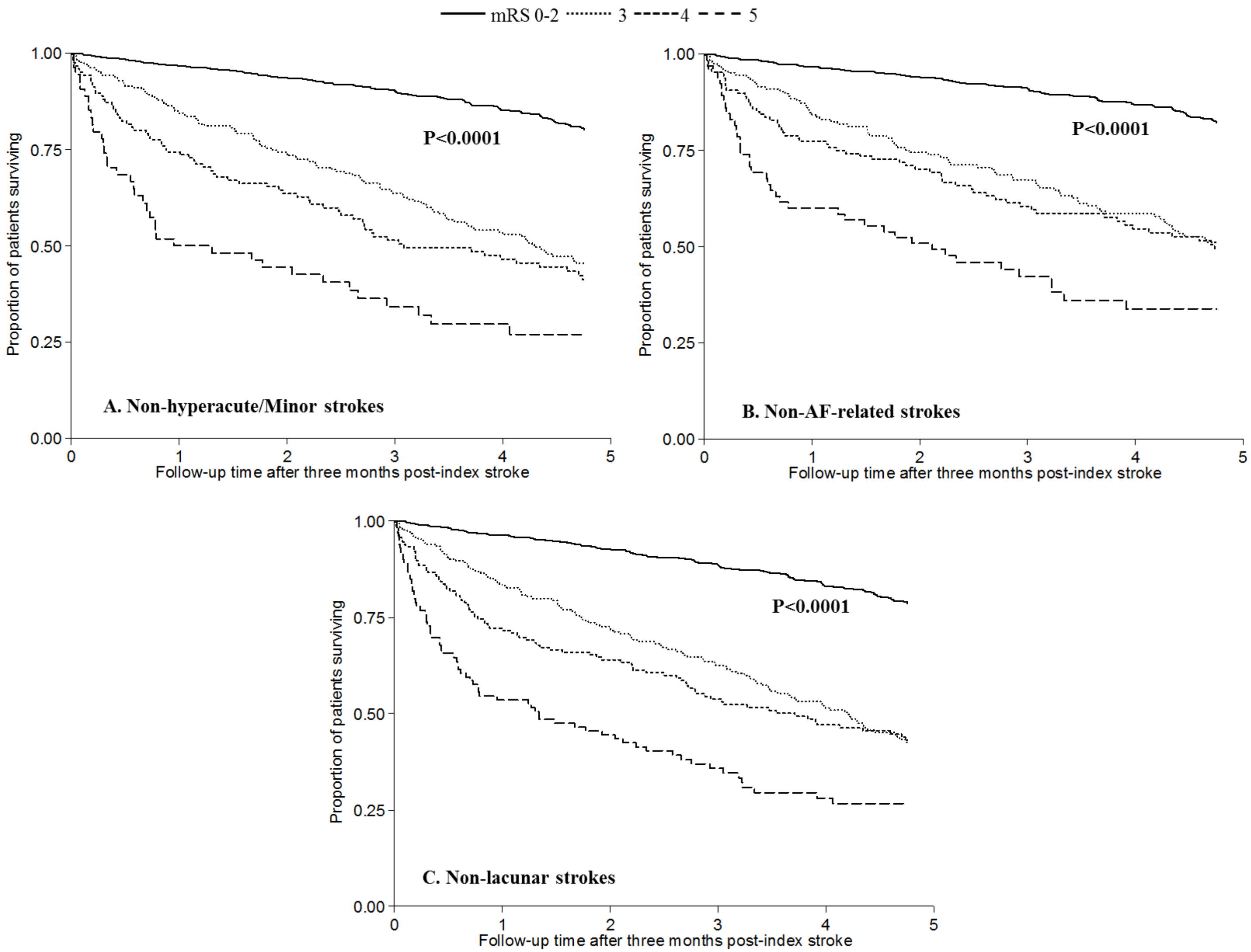


**Figure S4. Kaplan-Meier survival curves after index stroke for 3-month survivors (mRS 0-2 versus 3, 4, 5), for (A) non-hyperacute/minor strokes, (B) non-AF-related strokes, and (C) non-lacunar stroke subgroups.** P values obtained from the log-rank test for each group are displayed.

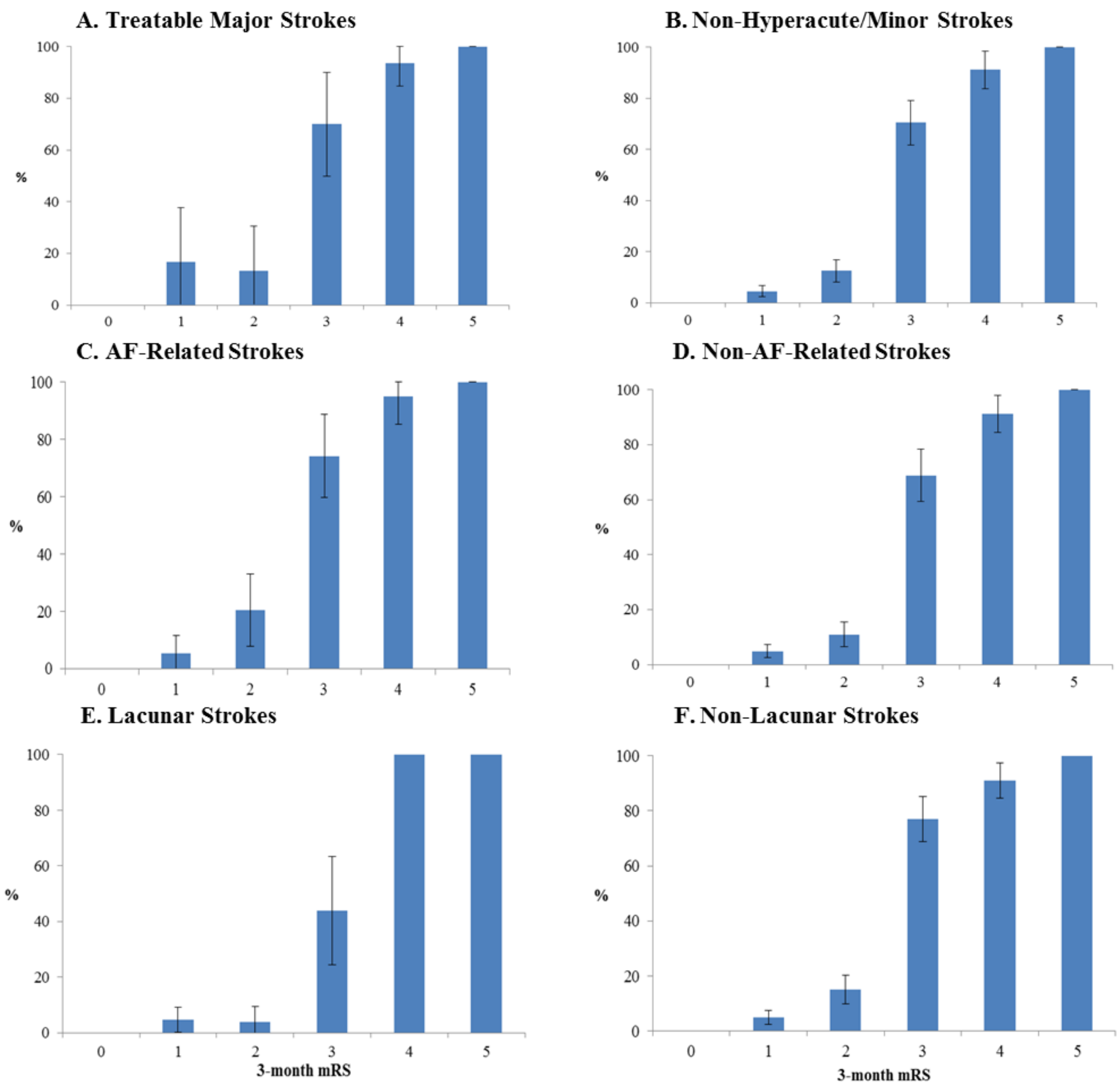


**Figure S5. Proportions of 3-month survivors of ischaemic stroke for key subgroups, stratified by pre-3-month mRS, who were disabled (mRS >2) at 5 years, only including those alive at 5 years in the total. Bars represent 95% confidence intervals.**

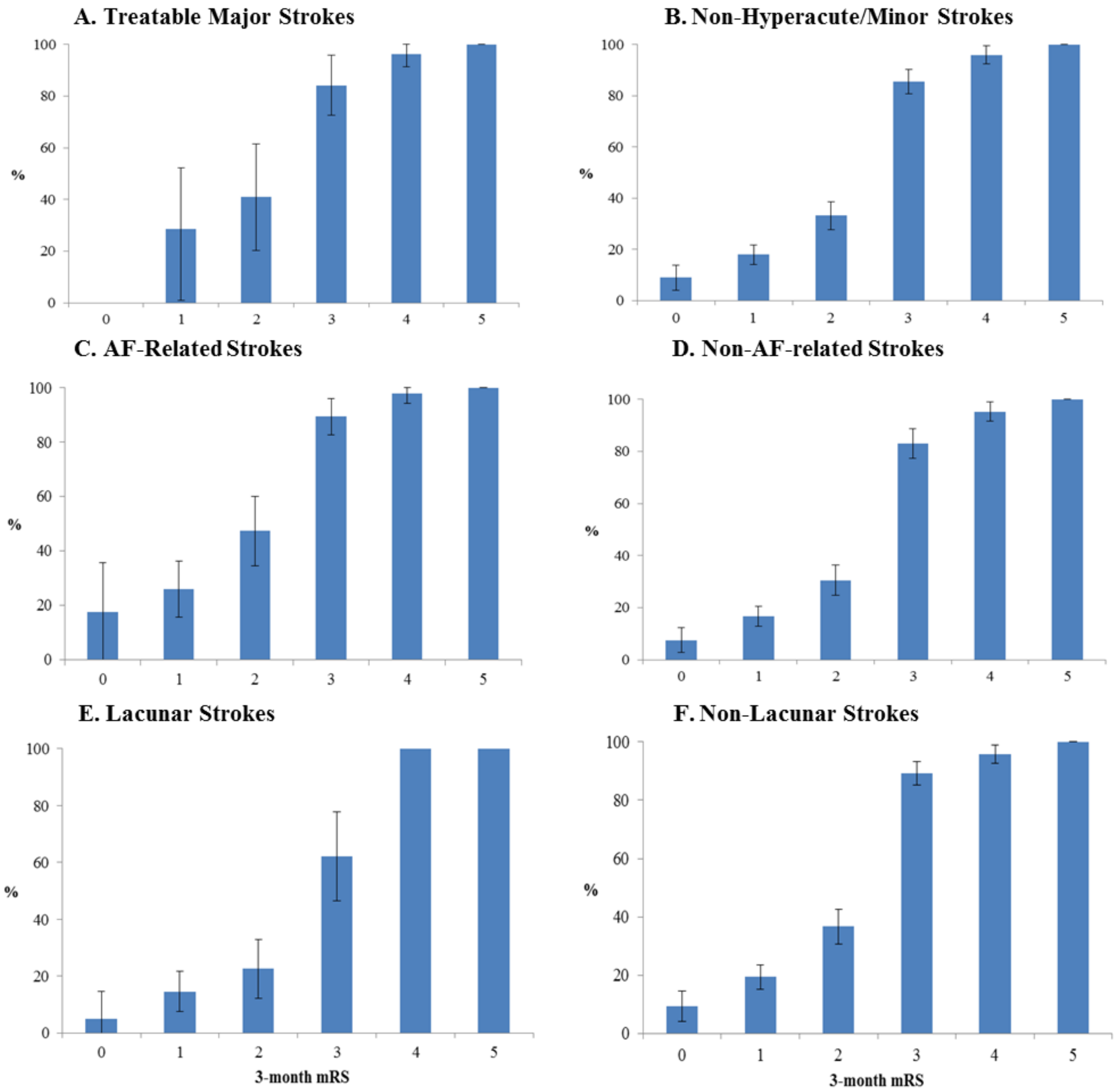


**Figure S6. Proportions of 3-month survivors of ischaemic stroke for key subgroups, stratified by pre-3-month mRS, who were dead or disabled at 5 years. Bars represent 95% confidence intervals.**
